# Supplementary material for: An Integrated Model including the ROX Index to Predict the Success of High-Flow Nasal Cannula Use after Planned Extubation: A Retrospective Observational Cohort Study
Source: J Clin Med. 2021 Aug 10;10(16):3513. doi: 10.3390/jcm10163513 (PMC8397019; doi:10.3390/jcm10163513)
Supplement: Supplementary file 1 [file jcm-10-03513-s001.zip › jcm-1309777-supplementary.pptx]

## Slide 1
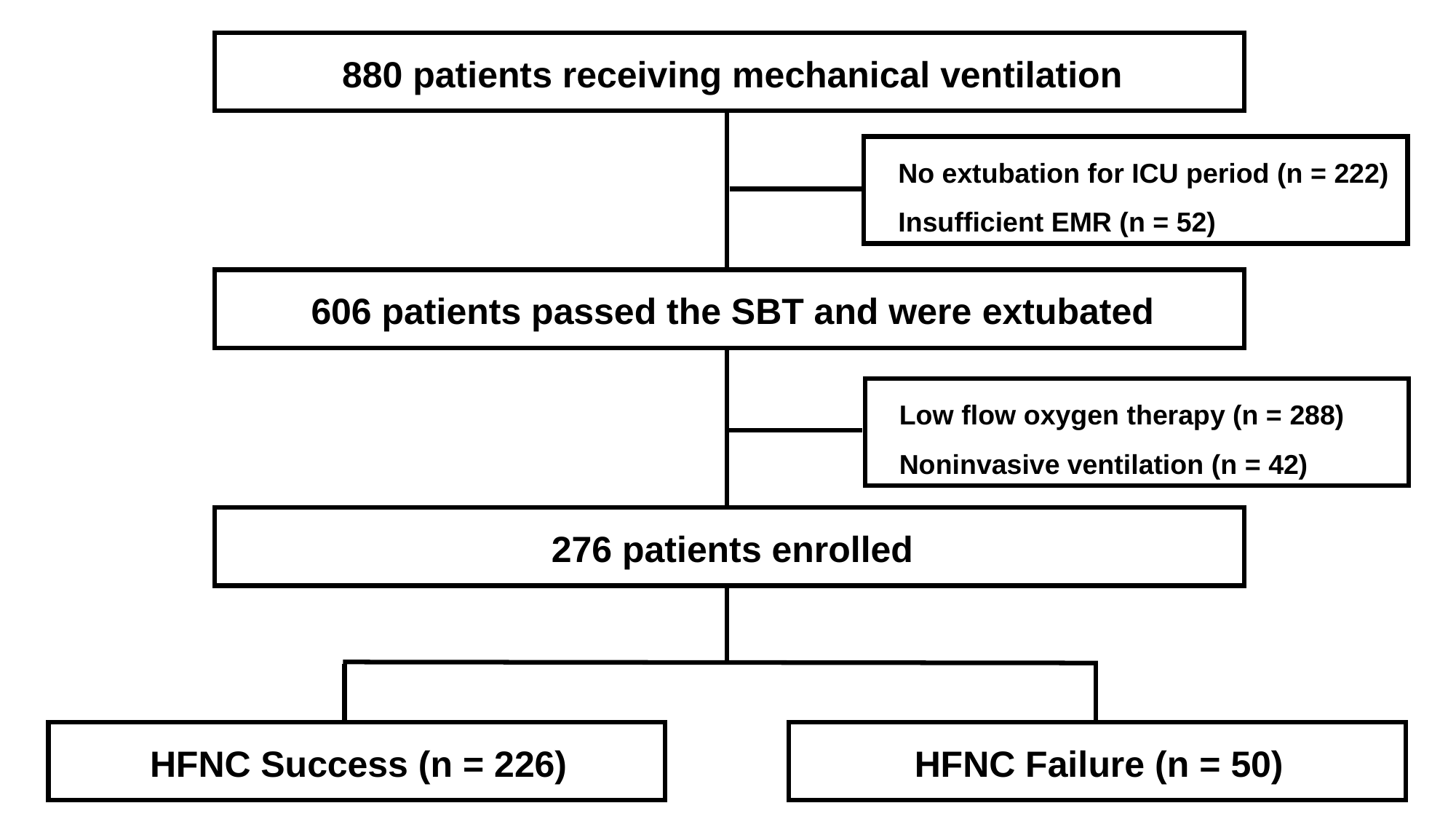

880 patients receiving mechanical ventilation
No extubation for ICU period (n = 222)
Insufficient EMR (n = 52)
606 patients passed the SBT and were extubated
Low flow oxygen therapy (n = 288)
Noninvasive ventilation (n = 42)
276 patients enrolled
HFNC Failure (n = 50)
HFNC Success (n = 226)

## Slide 2
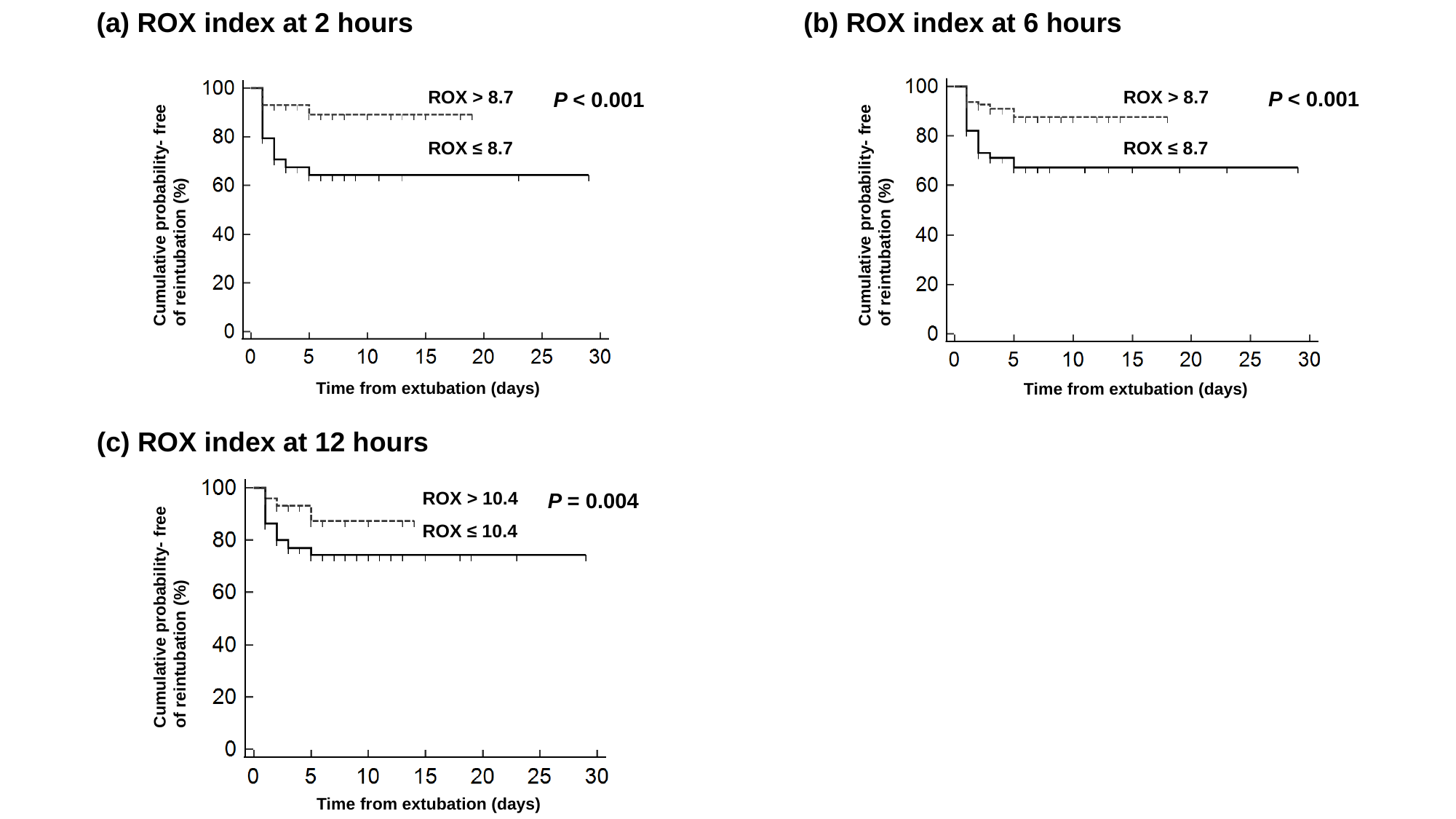

(a) ROX index at 2 hours
(b) ROX index at 6 hours
Cumulative probability- free of reintubation (%)
ROX > 8.7
P < 0.001
ROX ≤ 8.7
Time from extubation (days)
Cumulative probability- free of reintubation (%)
ROX > 8.7
P < 0.001
ROX ≤ 8.7
Time from extubation (days)
(c) ROX index at 12 hours
ROX > 10.4
Cumulative probability- free of reintubation (%)
P = 0.004
ROX ≤ 10.4
Time from extubation (days)

## Slide 3
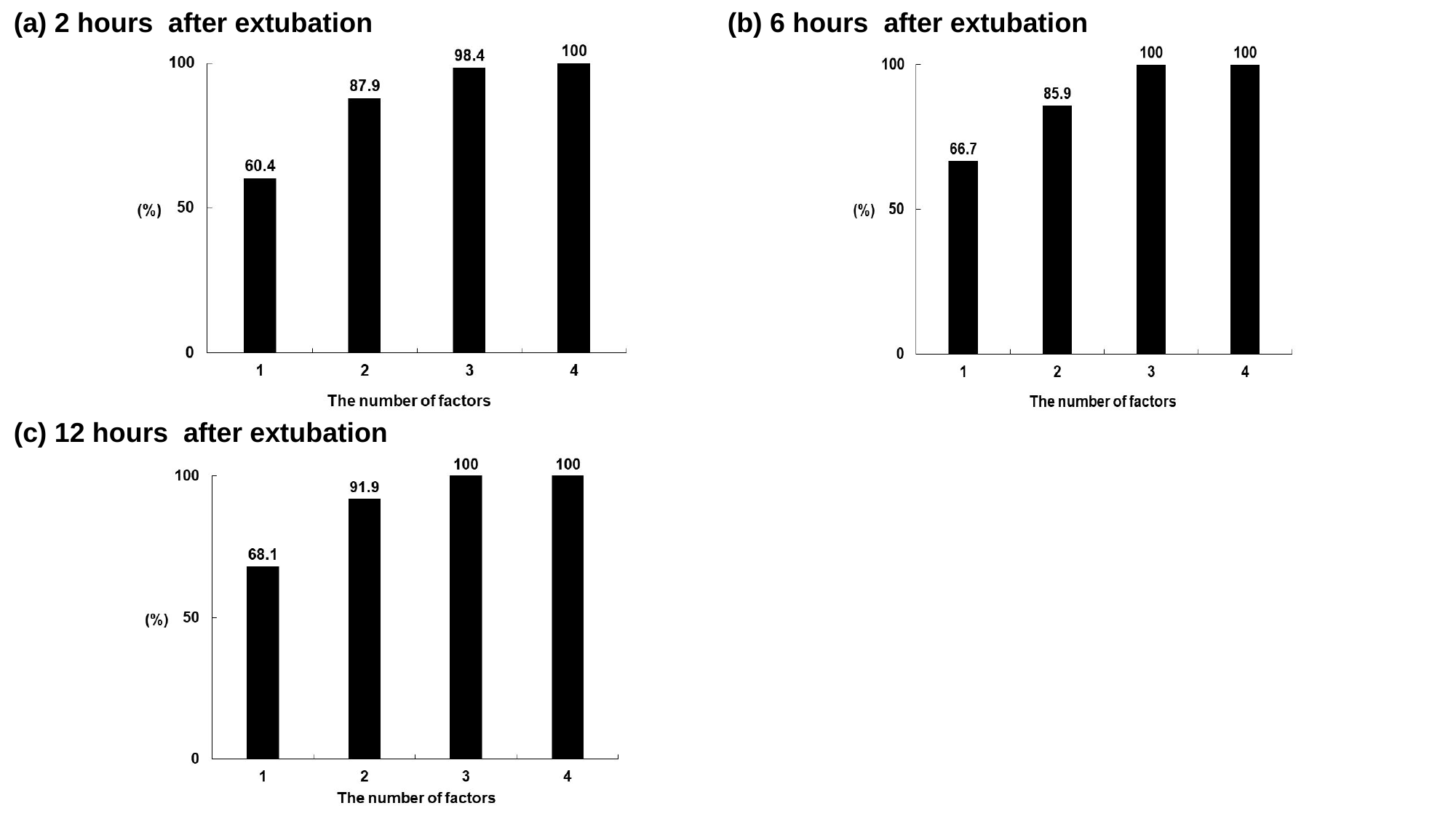

(a) 2 hours after extubation
(b) 6 hours after extubation
(c) 12 hours after extubation

## Slide 4
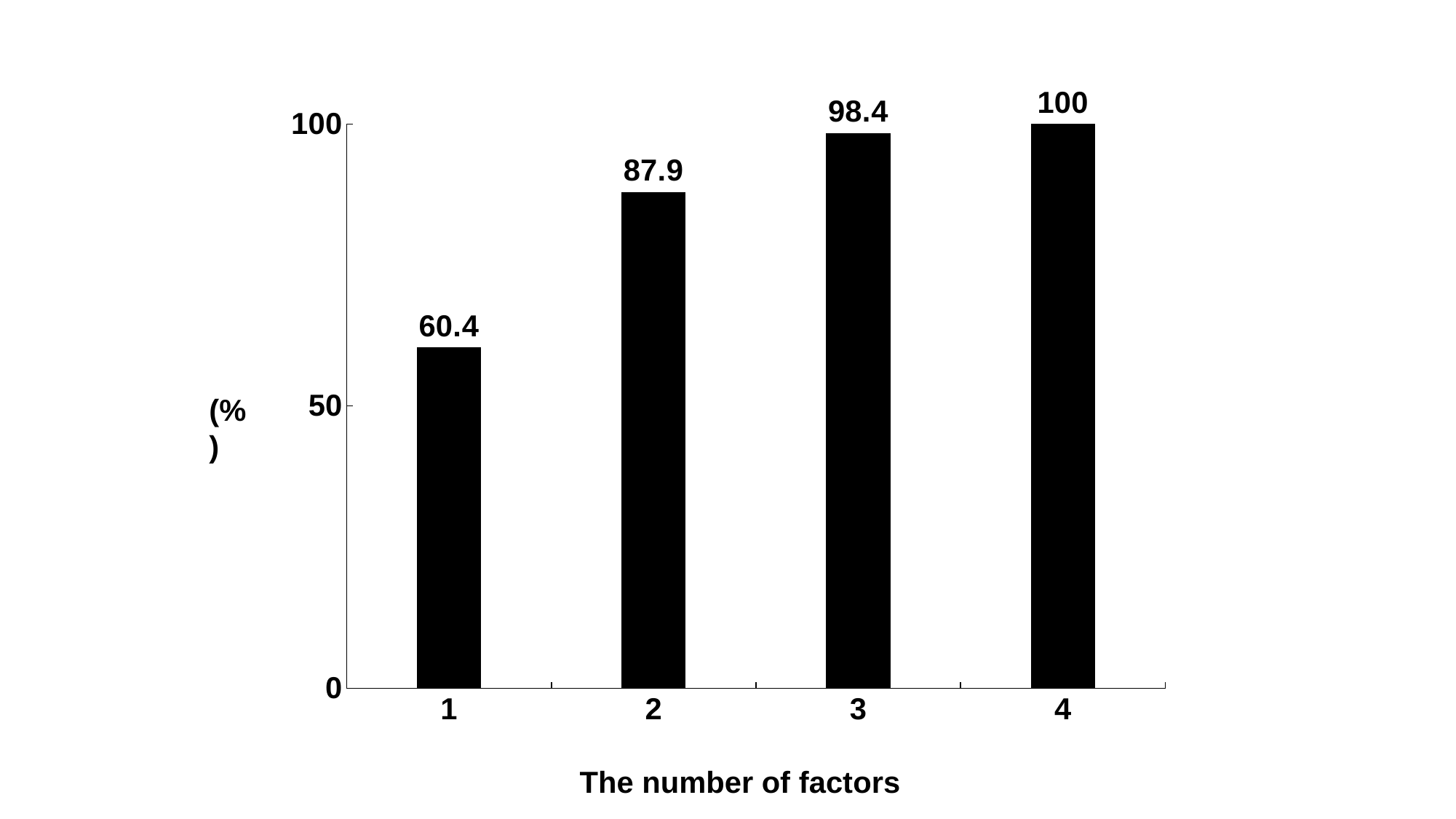

### Chart
| Category | |
|---|---|(%)
The number of factors

## Slide 5
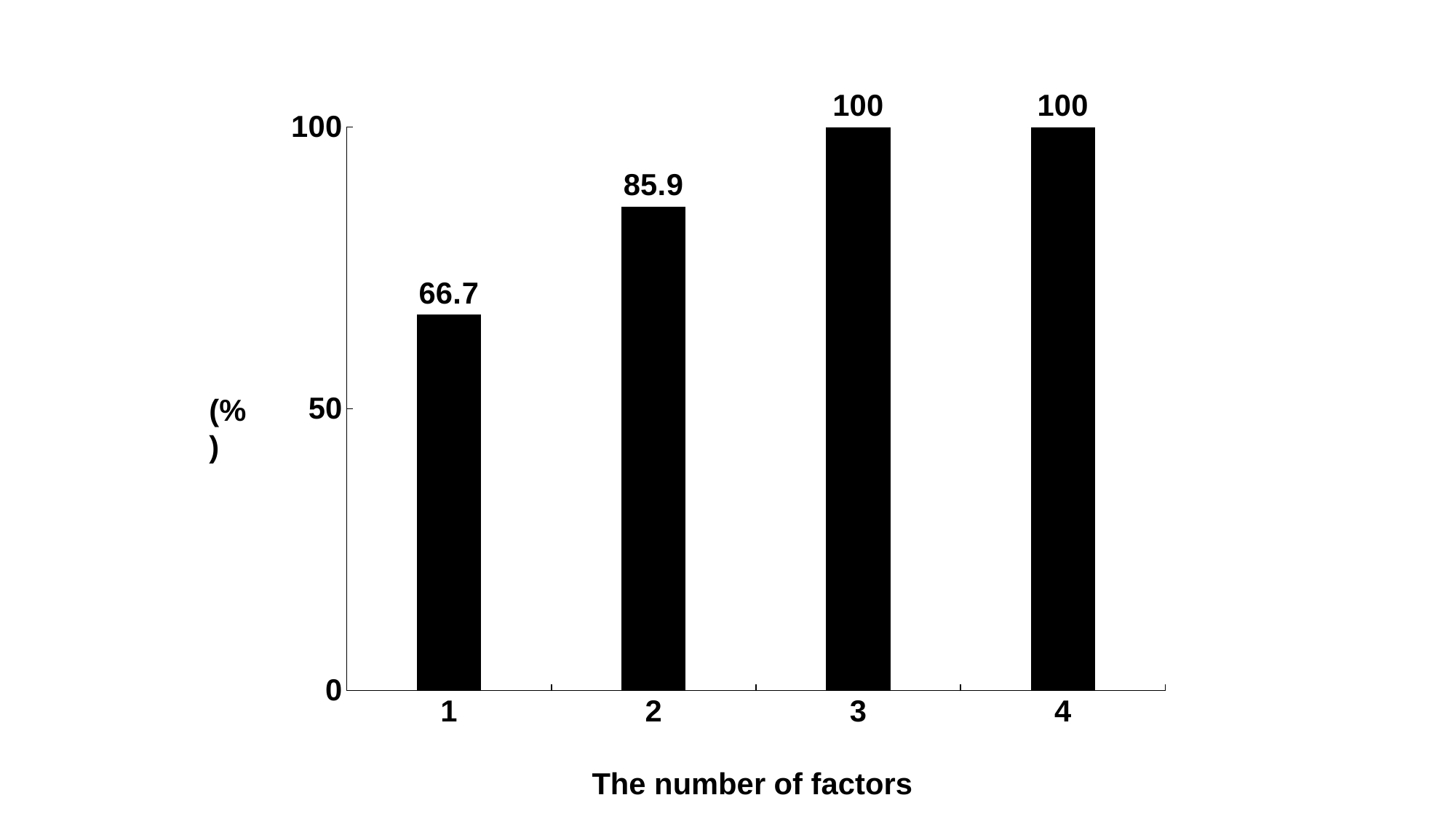

### Chart
| Category | |
|---|---|(%)
The number of factors

## Slide 6
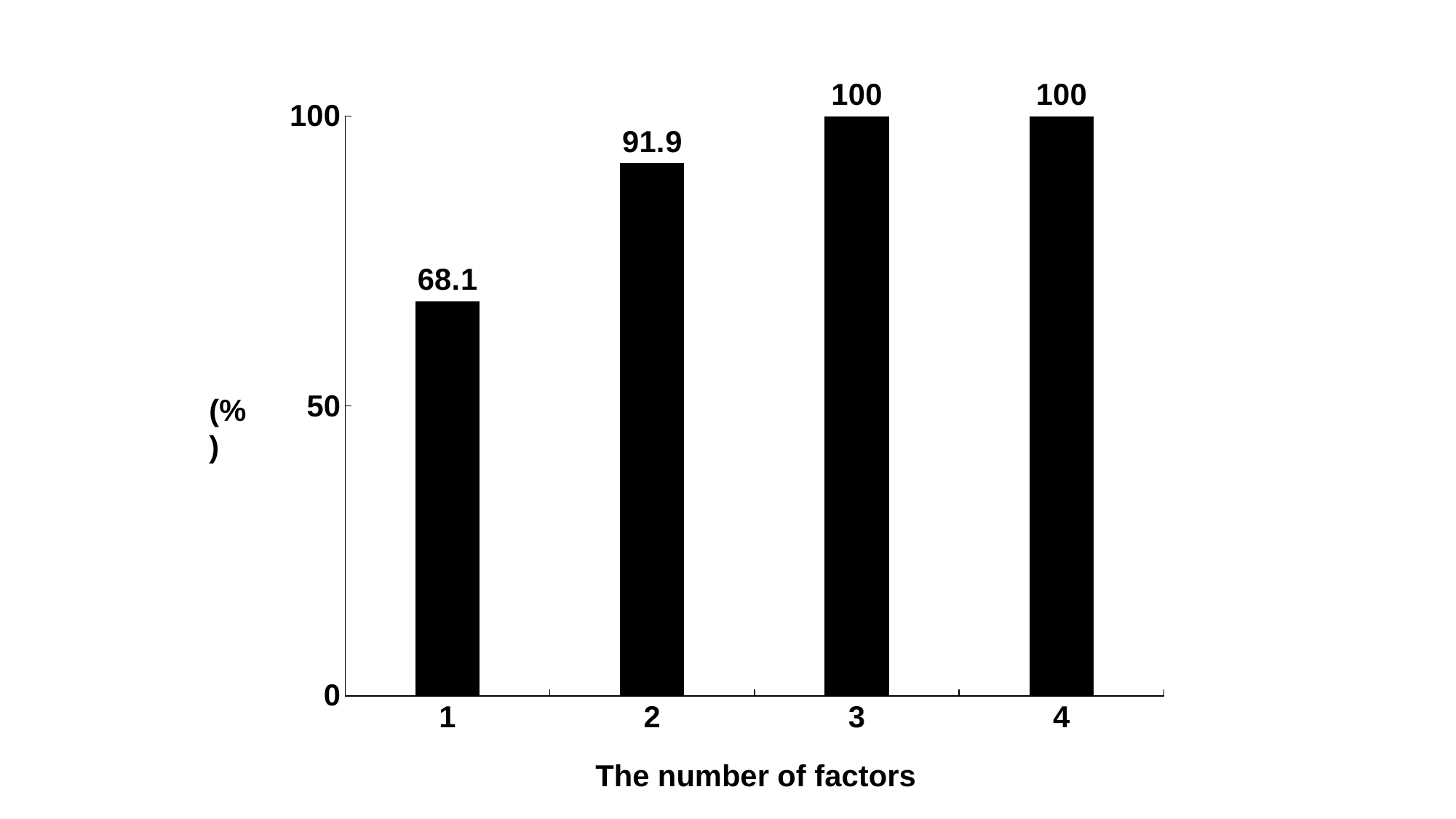

### Chart
| Category | |
|---|---|(%)
The number of factors
